# Innovations in clinical PET image reconstruction: advances in Bayesian penalized likelihood algorithm and deep learning

**DOI:** 10.1007/s12149-025-02088-7

**Published:** 2025-07-18

**Authors:** Kenta Miwa, Tensho Yamao, Fumio Hashimoto, Noriaki Miyaji, Yuto Kamitaka, Masaki Masubuchi, Taisuke Murata, Tokiya Yoshii, Rinya Kobayashi, Shohei Fukuda, Naochika Akiya, Kaito Wachi, Kei Wagatsuma

**Affiliations:** 1 Department of Radiological Sciences, School of Health Sciences, Fukushima Medical University, 10-6 Sakaemachi, Fukushima-Shi, Fukushima, 960-8516 Japan; 2 J. Crayton Pruitt Family Department of Biomedical Engineering, University of Florida, Gainesville, FL USA; 3 Department of Integrated Health Sciences, Nagoya University Graduate School of Medicine, Tokai National Education and Research System, Aichi, Japan; 4 Department of Radiology, University of Tsukuba Hospital, Ibaraki, Japan; 5 Department of Radiology, Chiba University Hospital, Chiba, Japan; 6 Department of Radiology, Fukushima Medical University Hospital, Fukushima, Japan; 7 Department of Radiological Technology, Tokai University Hospital, Kanagawa, Japan; 8 Department of Radiological Technology, Tokai University Hachioji Hospital, Hachioji, Tokyo Japan; 9 Faculty of Health Sciences, Hokkaido University, Hokkaido, Japan

**Keywords:** Artificial intelligence, Deep learning, Image reconstruction, Positron emission tomography

## Abstract

Recent advances in PET image reconstruction have focused on achieving high image quality and quantitative accuracy. Bayesian penalized likelihood (BPL) algorithms, such as Q.Clear and HYPER Iterative that have been integrated into commercial PET systems offer robust image noise suppression and edge preservation through regularization. In parallel, methods based on deep learning such as SubtlePET, AiCE, uAI^®^ HYPER DLR, and Precision DL have emerged primarily as post-processing techniques. They use trained convolutional neural networks to reduce image noise while preserving lesion contrast. These methods have reduced image acquisition times or reduced radiotracer doses while maintaining diagnostic confidence. uAI^®^ HYPER DPR represents a hybrid approach by embedding deep learning in iterative reconstruction. This review summarizes the technical principles and the clinical performance of BPL and deep learning-based PET reconstruction algorithms, and discusses key considerations such as image quality and quantitative accuracy of PET images. This review should deepen understanding of advanced PET image reconstruction techniques and accelerate their clinical implementation across diverse PET imaging applications.

## Introduction

**Fig. 1:**
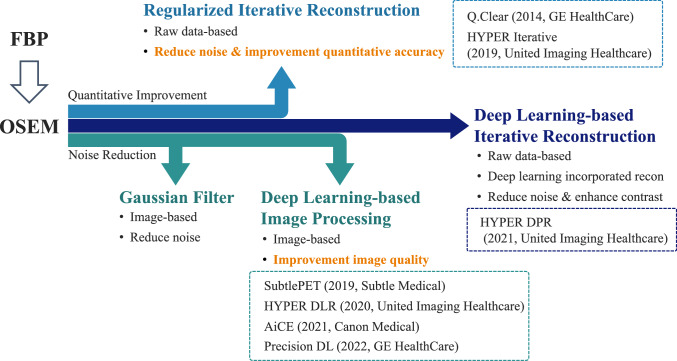
Evolution of commercial advanced PET image reconstruction. *BPL* Bayesian penalized likelihood, *DLR* deep-learning reconstruction, *FBP* filtered back-projection, *OSEM* ordered subset expectation maximization

Positron emission tomography (PET) has become an indispensable imaging modality in the diagnosis and treatment of cancer and neurodegenerative diseases such as dementia [1–4]. Recent advances in PET imaging have stressed the need to balance superior image quality that is critical for the sensitive detection of small lesions, and superior quantitative accuracy that is essential for repeatability and reproducibility [5–7]. Advanced PET image reconstruction methods play key roles in meeting these dual requirements (Fig. 1) [8–10].

Among the cutting-edge approaches to reconstruction, methods based on Bayesian penalized likelihood (BPL) enhance image quality while preserving quantitative accuracy [11]. By incorporating regularization techniques, methods such as block sequential regularized expectation maximization (BSREM), marketed as Q.Clear (GE HealthCare, Waukesha, WI, USA), and total variation regularized expectation maximization (TVREM), marketed as HYPER Iterative (United Imaging Healthcare, Shanghai, China), have been integrated into commercial PET/computed tomography (CT) and magnetic resonance imaging (MRI) systems. These methods use regularization to control image noise and preserve sharp edges, indicating their potential as standards in clinical PET image reconstruction.

New paradigms of deep learning-based PET reconstruction have enhanced PET imaging via post-processing and iterative methods that have helped to reduce image noise, improve spatial resolution, and maintain or enhance quantitative accuracy [12–14]. Post-processing techniques such as SubtlePET (Subtle Medical, Menlo Park, CA, USA), AiCE (Canon Medical Systems, Otawara, Japan), uAI^®^ HYPER DLR (HYPER DLR; United Imaging Healthcare), and Precision DL (GE HealthCare) focus on refining reconstructed PET images. SubtlePET, AiCE, and uAI^®^ HYPER DLR are trained on paired low- and high-quality PET images to suppress image noise while preserving critical diagnostic details. Precision DL learns differences between images with and without time-of-flight (TOF) PET. This has enhanced the image quality of non-TOF datasets. Among deep learning-based reconstruction methods, uAI^®^ HYPER DPR (HYPER DPR; United Imaging Healthcare) adopts a distinctive iterative approach. Unlike post-processing techniques, HYPER DPR generates reconstructed images by integrating deep learning models for image noise reduction and contrast enhancement within an iterative framework that allows HYPER DPR to refine images to achieve a balance between improved image quality and robust quantitative reliability. Such innovations (including post-processing and iterative deep learning-based methods) have verified the transformative impact of artificial intelligence (AI) on PET imaging having improved small lesion detection, and optimized imaging protocols that have improved clinical outcomes.

This review summarizes the technical aspects of BPL and deep learning-based reconstruction techniques, emphasizing their contributions to enhanced image quality and the quantitative accuracy of PET imaging.

### Basic principles of BPL reconstruction

Statistical iterative reconstruction methods based on maximum likelihood estimation such as ordered subsets expectation maximization (OSEM), have helped to significantly improved PET/CT image quality compared to analytical reconstruction methods such as filtered back projection (FBP). These improvements are largely due to incorporating accurate image noise modeling based on Poisson statistics and physical resolution modeling for detector response characteristics [15, 16]. However, the statistical noise in the data dominates image quality and worsens as the numbers of iterations increase. Therefore, the number of iterations in OSEM to prevent image noise amplification is limited, and post-filters are applied to smooth reconstructed images. The convergence of OSEM also depends on the shape and activity distribution of the target, complicating the determination of optimal parameters while balancing the trade-off between spatial resolution and image noise. Reconstruction using BPL algorithms is based on maximum a posteriori (MAP) estimations that have addressed these challenges by integrating prior information about target images using Bayesian inference. The likelihood of the data is combined with a prior term to maximize the posterior probability. Constraints designed to promote smoother PET images are incorporated as penalty functions that mitigate the enhanced image noise in OSEM, especially in later iterations. Penalty functions suppress excessive variations between neighboring pixels by regularization, which is achieved by adjusting values toward smoother directions. Regularization is a central feature of BPL methods that optimize the trade-off between spatial resolution and image noise. Incorporating advanced regularization techniques into PET/CT and PET/MRI systems has improved the performance of BPL methods such as BSREM (Q.Clear) and TVREM (HYPER Iterative). These robust tools have become established for improving image quality while maintaining quantitative accuracy.

## Advanced reconstruction algorithms using BPL reconstruction

### Q.Clear (BSREM)

#### Principles of Q.Clear

The first commercial PET/CT system to incorporate BPL reconstruction was Q.Clear [17], which adjusts the BPL objective function using numerical algorithms to achieve convergence. A relative difference penalty (RDP), introduced by Nuyts et al. [17, 18], is also embedded in the BPL objective function to control image quality during reconstruction.

The BPL objective function in Q.Clear is defined as [19]

1 $$\phi \left( x \right) = \mathop \sum \limits_{i} y_{i} \log \left( {\left[ {Px} \right]_{i} + r_{i} } \right) - \left( {\left[ {Px} \right]_{i} + r_{i} } \right) - \beta R\left( x \right)_{,}$$

where *x* is a voxel of interest, *y*_*i*_ is emission sinogram data, *P* is the system matrix (accounting for attenuation correction, normalization, and point spread function (PSF) correction), *r*_*i*_  is the estimated background distribution for scatter and random events, *R(x)* is the regularization or penalty function, and *β* is a weighting factor that determines the overall degree of regularization.

The RDP function is expressed as [18]

2 $$R\left( x \right) = \mathop \sum \limits_{j} \mathop \sum \limits_{{k \in N_{j} }} w_{jk} \sqrt {\beta_{j} \beta_{k} } \frac{{\left( {x_{j} - x_{k} } \right)^{2} }}{{x_{j} + x_{k} + \gamma \left| {x_{j} - x_{k} } \right|}},$$

where* N*_*j*_ represents the set of voxels adjacent to voxel *j*, *W*_*jk*_ is a distance-dependent weighting factor, *β*_*j*_ is the penalty modulation coefficient, and *γ* is a parameter that adjusts the degree of edge preservation. The RDP is a convex function that applies adaptive smoothing based on the activity concentration of the target region [20]. It imposes more and less smoothing in regions of lower and higher activities, respectively. This approach balances image noise reduction in background regions and preserves the edges of hot lesions, addressing a traditionally challenging trade-off in PET image reconstruction. Increasing the value of the γ factor enhances quantitative accuracy and mitigates the partial volume effect on small lesions [21]. However, a larger γ factor (γ ≥ 5) might introduce undesirable visual blocky and patchy artifacts in reconstructed PET images [17]. In contrast, a smaller γ factor (γ ≤ 1) somewhat improves quantitation. Considering the trade-off between visual image quality and small lesion quantitation, Q.Clear has a default γ factor of 2 [17, 22]. Q.Clear uses BSREM as a numerical optimizer to calculate images reconstructed using the BPL by maximizing its objective function [22–24]. The optimizer ensures convergence of the objective function while simultaneously suppressing image noise amplification through the RDP penalty function [23, 24]. Hence, Q.Clear is often referred to as BSREM.

**Fig. 2:**
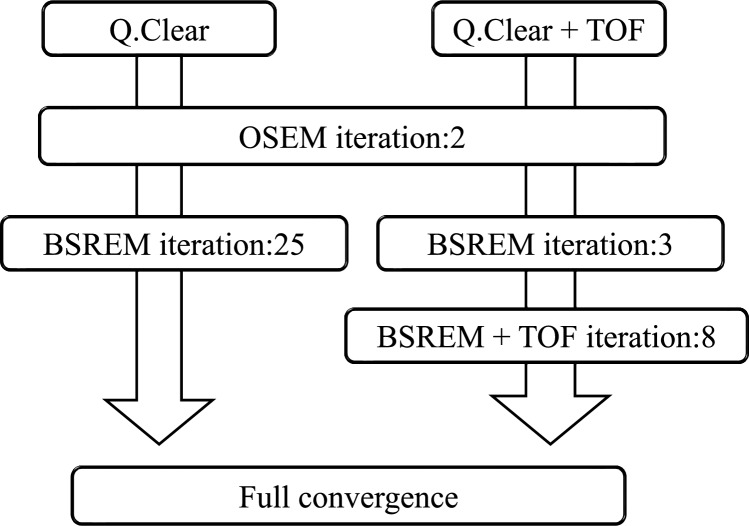
Internal processing flow differs between Q.Clear and Q.Clear + TOF. *BSREM* block sequential regularized expectation maximization, *OSEM* ordered subset expectation maximization, *TOF* time of flight

Q.Clear can be optionally combined with TOF reconstruction. However, the internal reconstruction conditions differ between Q.Clear alone or with TOF. Figure 2 shows the internal workflow of these reconstruction methods. Q.Clear combined with TOF (Q.Clear + TOF) improves convergence speed, which leads to a reduction in the number of BSREM updates to minimize image noise amplification.

#### Optimization of β value in Q.Clear

The penalization factor β is the only adjustable parameter in Q.Clear, and its careful optimization is fundamental to balancing image noise, contrast, and quantitative accuracy [25–27]. The findings of phantom studies have shown that increasing β reduces image noise while decreasing image contrast, emphasizing the need for case-specific adjustment [22, 28, 29]. The optimal β value varies depending on clinical objectives, imaging regions, target-to-background ratios (TBR) of lesions, radiotracer characteristics, and the amount of time needed to acquire images [27, 30, 31]. By tailoring the β value, Q.Clear can achieve high quantitative accuracy with superior signal-to-noise ratio (SNR) and signal-to-background ratio (SBR), even at noise levels comparable to those of OSEM [32]. This adaptability allows for significantly reduced injected radiotracer doses or acquisition durations, often by 50%–66% relative to OSEM [33–36]. The β values optimized for a wide range of radiotracers and clinical applications, highlight their influence on image quality and quantitation. These tracer- and application-specific optimal β values, as identified in prior clinical and phantom-based studies, are summarized in Table 1. The β values should be optimized considering image quality, lesion detectability, quantitative accuracy, clinical objectives, and acquisition duration [37]. By incorporating insights from global studies, institutions can refine their β settings to suit specific clinical and research needs [31, 38].

**Table 1:** Summary of optimized β values for Q.Clear in various PET imaging settings

Publication	Tracer	Application	Optimal β value	Scanner type	Study type
Teoh et al. 2015 [22]	^18^F-FDG	Pulmonary nodules	400	Discovery 690 PET/CT	Phantom and clinical
Howard et al. 2017 [39]	^18^F-FDG	Small pulmonary nodules (mean diameter of 8 mm)	150	Discovery IQ PET/CT	Clinical
Wangerin et al. 2017 [40]	^18^F-FDG	Synthetic spherical lesions in liver and lung regions	450 (liver); 250 (lung)	Discovery 690 PET/CT	Clinical
Rowley et al. 2017 [41]	^90^Y	Post- selective internal radiotherapy (SIRT) imaging for the treatment of liver malignancies	4,000	Discovery 710 PET/CT	Phantom and clinical
O’Doherty et al. 2017 [42]	^13^N-NH_3_	Quantitative myocardial blood flow (MBF) imaging	300	Discovery 710 PET/CT	Clinical
Reynes-Llompart et al. 2018 [27]	^18^F-FDG	Brain acquisitions (neuro-oncologic) and torso acquisitions (oncologic whole body acquisition)	200 (brain); 350 (torso)	Discovery IQ PET/CT	Phantom and clinical
Teoh et al. 2018 [43]	^18^F-fluciclovine	Prostate cancer	300	Discovery 710 PET/CT	Clinical
Bjöersdorff et al. 2019 [44]	^18^F-fluorocholine	Prostate cancer	400–550	Discovery MI PET/CT	Clinical
Miwa et al. 2020 [45]	^18^F-FDG	Sub-centimeter tumor lesions	200	Discovery MI PET/CT	Phantom
Lindstrom et al. 2020 [46]	^18^F-flutemetamol	Alzheimer’s disease (AD) with Aβ pathology	300	Discovery MI PET/CT	Clinical
Texte et al. 2020 [47]	^18^F-FMISO/^18^F-FAZA	Hypoxia imaging of non-small cell lung carcinoma (NSCLC)	350	Discovery 710 PET/CT	Phantom and clinical
Seo et al. 2020 [48]	^68^ Ga-citrate	Prostate cancer with castration-resistant disease	500	SIGNA PET/MRI	Phantom and clinical
Yoshii et al. 2020 [34]	^18^F-NaF	Bone metastases	400	Discovery MI PET/CT	Phantom and clinical
Baratto et al. 2020 [49]	^68^ Ga-RM2/^68^ Ga-PSMA	Prostate cancer	400–600	SIGNA PET/MRI	Clinical
Witkowska-Patena et al. 2020 [50]	^18^F-PSMA-1007	Prostate cancer	350	Discovery 710 PET/CT	Clinical
Chicheportiche et al. 2021 [33]	^68^ Ga-DOTA	Neuroendocrine tumors	1,300–1,600	Discovery MI PET/CT	Phantom and clinical
Kirchner et al. 2021 [51]	^89^Zr-immunoPET tracer	^89^Zr-immuno PET imaging	3,600	Discovery 710 PET/CT	Clinical
Usmani et al. 2021 [52]	^18^F-NaF	Skeletal staging in obese patients	600	Discovery 710 PET/CT	Phantom and clinical
Rijnsdorp et al. 2021[53]	^68^ Ga-PSMA	Prostate cancer	600	Discovery 710 PET/CT	Clinical
Wagatsuma et al., 2022 [25]	^11^C-PiB/^18^F-FDG	Alzheimer’s disease (AD) with Aβ pathology	300 (^11^C-PiB); 200 (^18^F-FDG)	Discovery 710 PET/CT	Phantom and clinical
Ribeiro et al. 2022 [54]	^11^C-PHNO	Dynamic brain PET	100–200	SIGNA PET/MRI	Clinical
Tian et al. 2022 [55]	^18^F-FDG	General tumor PET/MRI imaging	400	SIGNA PET/MRI	Phantom and clinical
Ruan et al. 2023 [36]	^68^ Ga-FAPI	Lesions with obviously high FAPI uptake	350	SIGNA PET/MRI	Clinical
Margail et al. 2023 [56]	^68^ Ga-PSMA-11	Biochemical recurrence of prostate cancer	400–600	Discovery 710 PET/CT and Discovery MI PET/CT	Clinical
Young et al. 2023 [57]	^15^O-H_2_O	Dynamic PET for quantitative cerebral blood flow (CBF) measurements	300	Discovery MI PET/CT	Clinical
Sadeghi et al. 2023 [58]	^18^F-FDG	Rectal cancer detection using BGO-based PET	500 (large lesions); 300(small lesions)	Discovery IQ PET/CT	Phantom and clinical
Fukuda et al. 2024 [59]	^18^F-flutemetamol	Amyloid PET imaging without PSF correction	300	Discovery MI PET/CT	Phantom and clinical
Fooladi et al. 2024 [37]	^68^ Ga-PSMA	Robustness of radiomics features in PSMA-PET images	300–500	Discovery IQ PET/CT	Phantom and clinical
Wagatsuma et al. 2024 [60]	^18^F-flutemetamol	Amyloid PET imaging without PSF correction	600	Discovery MI PET/CT	Phantom and clinical
Calatayud-Jordán et al. 2024 [61]	^90^Y	^90^Y PET/MR imaging after SIRT	4,000 (high image quality); 1,500–2,000 (high quantitation)	SIGNA PET/MRI	Phantom
Di Franco et al.\, 2024 [62]	^68^ Ga-DOTANOC	Neuroendocrine tumors	1,300–1,600	Discovery MI PET/CT	Clinical
Bonney et al. 2025 [63]	^89^Zr	Optimizing phantom studies for quantitative imaging	1,500	Discovery 710 PET/CT	Phantom

#### Key technical aspects of Q.Clear: physical evaluation of image quality, SNR, and SUV

The established technical advantages of Q.Clear are better image quality while maintaining superior quantitation accuracy and SNR for lesions compared with OSEM [17, 22, 64, 65]. Furthermore, the standardized uptake values (SUVs) of lesions are higher and more accurate for Q.Clear than OSEM [22, 64, 66]. The ability of Q.Clear to differentiate malignant from benign lung nodules using SUV_max_ has significantly improved the diagnostic accuracy of sub-centimeter lesions (< 10 mm) compared with OSEM [64]. An investigation of liver metastases of rectal cancer has confirmed that Q.Clear selectively increases the SUV of metastatic lesions without affecting the SUV or levels of noise in the liver background [66]. The performance of OSEM is poor in cold region due to convergence limitation. In contrast, the incorporated edge-preserving effects of the RDP in Q.Clear delivers superior quantitation accuracy in cold background area, such as lung [17, 21, 40]. However, Q.Clear might overestimate PET values under some clinical conditions [67]. For example, using Q.Clear to diagnose lymphoma using Deauville scores is challenging [68–71]. Regardless, Q.Clear has substantially improved the quality of images acquired from patients who are overweight [72–75]. This is particularly significant for patients with a high body mass index (BMI), and of elevated background image noise that renders the detection and quantitation of small abnormalities especially challenging [11, 72]. In contrast, Q.Clear has significantly improved liver SNR compared with OSEM in patients with high BMI [73]. The authors of these reports have recommended that the amount of activity administered should be linearly adjusted according to body weight to optimize Q.Clear performance.

#### Key technical aspects of Q.Clear: effects on visual lesion detectability

**Fig. 3:**
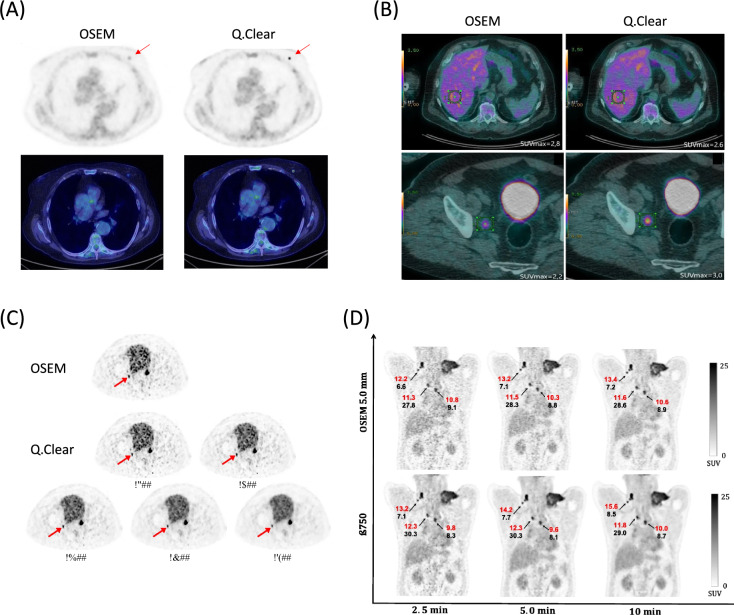
Comparison of lesion detectability between OSEM and Q.Clear. **A** Incidental findings of nodule in left mammary gland with SUVs of 1.8 and 5.0 determined by OSEM and Q.Clear, respectively. **B** Iliac lymph node uptake due to malignant lymphoma. Whereas liver SUVs are comparable, those of Q.Clear were higher than OSEM for iliac lymph nodes. **C** Ability of Q.Clear to detect small lymph nodes metastasized from prostate cancer improved owing to noise reduction of ^68^ Ga-PSMA PET images. **D** Red and black arrows respectively indicate SUV_mean_ and metabolic active tumor volume (MATV) of malignant lymphoma. Ability of Q.Clear to detect small lymph nodes is enhanced due to reduced image noise. (Reprinted with modification from the following publications: Aide et al. [11] (**A**), Wyrzykowski et al*.* [68] (**B**), Ter Voert et al*.* [79] (**C**), and Caribe et al*.* [77] (**D**). Copyright © 2021, Aide et al. 2020, Wyrzykowski et al. 2018, Ter Voert et al. 2019, and Caribe et al. licensed under CC BY 4.0)

Q.Clear delivers superior visual lesion detectability, particularly for small metastases in the lung and liver [22, 45, 76–78]. Figure 3 shows that the improved lesion detectability of Q.Clear is attributed to the edge-preserving effects of the RDP. The ability of Q.Clear to detect lesions is contrast-dependent [40]. Although the quantitative accuracy of Q.Clear is better for lung, than liver lesions [17], it can detect liver lesions in regions with higher contrast [40]. This is due to the powerful smoothing effect of RDP in regions of low activity, such as the lung. Higher contrast as further enhanced the ability of Q.Clear to detect small lesions. Q.Clear improves the spatial resolution and enhances the ability to detect lesions < 10 mm [28, 45, 78, 79]. However, like OSEM with post-filters, Q.Clear might cause small, low-contrast lesions to blend into the background, which warrants careful interpretation [40, 80]. Compared with OSEM, Q.Clear-based PET images improve the automated detection of small pulmonary nodules using deep learning [81]. In addition, combining Q.Clear with TOF further improves the detectability of even small lesions with low contrast, and liver, over lung lesions are favored [76]. These findings underscore the synergistic effect of these technologies [40]. Changes in lesion detectability with Q.Clear might affect the interpretation of PET images. For example, in ^18^F-FDG tumor PET imaging, Q.Clear can prominently visualize small, metabolically active structures that are less apparent with OSEM. The aortic walls of younger, healthy individuals is distinguishable from the blood pool, and accumulation in the adrenal glands and spinal cord also becomes more pronounced. Moreover, Q.Clear enhances small reactive lymph nodes in the neck and bilateral hilar or mediastinal lymph nodes associated with tumor-related sarcoid-like reactions [11]. Thus, healthcare professionals using Q.Clear-based PET imaging reconstruction require a specific level of practical experience and training to understand the unique characteristics of clinical PET images reconstructed using Q.Clear [82].

#### Technical limitations and challenges in Q.Clear: Q.Clear without PSF correction, γ factor adjustment, and application to SPECT

**Fig. 4:**
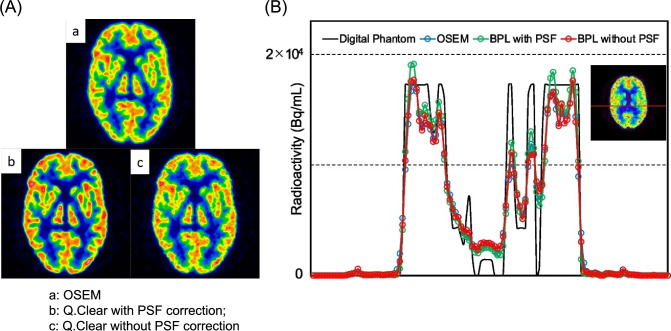
Images (**A**) and count profile curves (**B**) at nucleus basalis level in Hoffman 3D brain phantom under three reconstruction conditions. Gibbs artifacts revealed by Q.Clear disappear without PSF. *BPL* (Q.Clear) Bayesian penalized likelihood, *OSEM* ordered subset expectation maximization, *PSF* point spread function. (Reprinted with modification from Wagatsuma et al. [60], licensed under CC BY 4.0)

The PSF correction is automatically incorporated into Q.Clear [83]. This provides not only the benefits of improved convergence efficiency over the BPL method but also the enhanced contrast effects associated with PSF correction [22]. However, concerns have arisen regarding the potential for Gibbs artifacts (edge artifacts) associated with PSF correction [84, 85]. The RDP effect and appropriate adjustment of the β value in Q.Clear can mitigate these artifacts [29, 86]. As an alternative approach, we have been exploring the performance of Q.Clear without PSF correction in brain PET imaging. We found that Q.Clear without PSF correction fixed the unnatural enhancement of cortical uptake in the brain caused by Gibbs artifacts [60]. The image quality and quantitative values obtained from phantom images using Q.Clear without PSF correction were respectively equivalent to or better than those obtained using OSEM reconstruction, but were inferior to those obtained with Q.Clear with PSF correction. The optimal reconstruction conditions for Q.Clear without PSF correction further enhanced its potential as an alternative approach to reconstruction [59]. These findings suggest that Q.Clear without PSF correction could be a viable option for specific applications where minimizing the impact of artifacts is critical (Fig. 4).

Q.Clear currently allows users to modify only the β value, which adjusts the strength of the RDP. However, modifying the γ factor within the RDP formula can offer the potential to acquire PET images with higher spatial resolution [17]. We therefore evaluated the fundamental characteristics of the γ factor in Q.Clear using NEMA body phantoms and micro hollow spheres [21]. A lower γ factor in RDP works as a quadratic mode for smoothing, whereas a higher γ factor works as a linear mode for edge-preserving, thus enabling higher spatial resolution [87]. In another study, we identified the optimal reconstruction parameters for Q.Clear without PSF correction for ^18^F-flutemetamol amyloid PET images, which was a γ factor of 10, 1 iteration, and a β value of 800 [59].

Q.Clear has recently been implemented in the 360° ring-shaped CZT-based SPECT/CT system, StarGuide (GE HealthCare), extending its application beyond PET to SPECT imaging [88]. In StarGuide, Q.Clear provides flexibility in regularization, allowing the user to choose between configurations with no regularization or with regularization terms such as the RDP or the median root prior (MRP) [89]. When RDP regularization is used, as in this study, two parameters-the β value and the γ factor-can be adjusted to fine-tune the regularization process [90]. Danieli et al. evaluated the performance of the Q.Clear reconstruction algorithm for SPECT/CT and demonstrated its ability to accurately quantify ^177^Lu activity, with deviations within 10% in large volumes [90].

### HYPER Iterative (TVREM)

#### Principles of HYPER Iterative

HYPER Iterative incorporates a mathematical term to regulate image noise into the reconstruction process [91]. Although OSEM requires users to define the number of iterations, HYPER Iterative automatically continues iterations until they converge. This approach enables more accurate quantitation and delivers PET images with higher SNRs.

HYPER Iterative estimates reconstructed images by maximizing the BPL objective function (Eq. 3), which includes a penalty term *U(f)* to regulate image noise and the γ parameter that serves as a weighting factor to adjust the overall degree of the regularization. The type of penalty function in HYPER Iterative is total variation (TV) [92] that smooths only uniform regions while preserving sharp edges with large intensity gradients, ensuring enhanced edge preservation:

3 $$\hat{f} = argmax_{f \ge 0} \left[ {\mathop \sum \limits_{ij} - p_{ij} f_{j} + \mathop \sum \limits_{i} c_{i} \ln \left( {\mathop \sum \limits_{j} p_{ij} f_{j} } \right) - \mathop \sum \limits_{j} \gamma_{j} \times U\left( {f_{j} } \right)} \right]$$

4 $$\gamma_{j} = g\left( {NEC, sns_{j} } \right) \times \beta$$

5 $$U\left( f \right) = \mathop \sum \limits_{x,y,z} \left| {\nabla f} \right|$$

where $f$ is the estimated PET image, *i* and *j* are indices for projection bins and image voxels, respectively, ${c}_{i}$ is the measured emission data, and ${p}_{ij}$ is the system matrix (3);  ${\gamma }_{j}$ adjusts the noise level, *g* is a function that incorporates *NEC*, the noise equivalent count and *sns*_*j*_*, which* is the spatially varying sensitivity profile, $\beta$ is a normalized coefficient (ranging from 0 to 1) that determines the strength of the regularization or penalty function (4) and *U* is the TV penalty function applied to neighboring voxels (5).

**Fig. 5:**
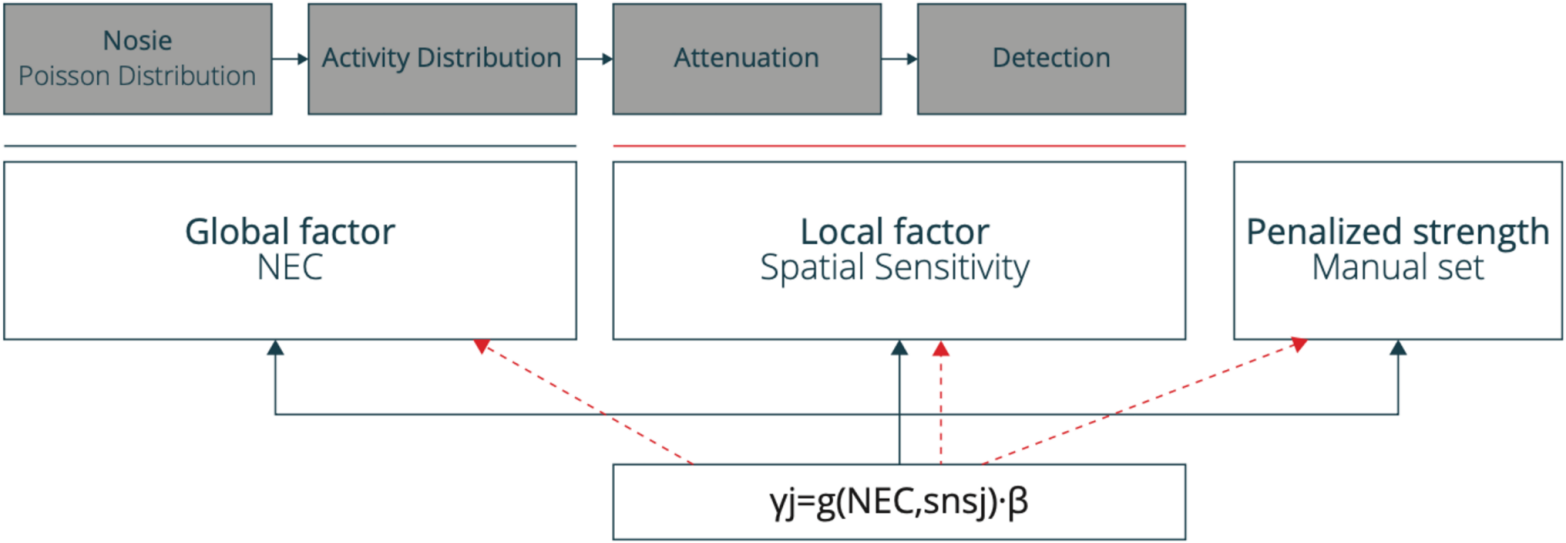
Modeling the γ parameter in HYPER Iterative reconstruction

**Fig. 6:**
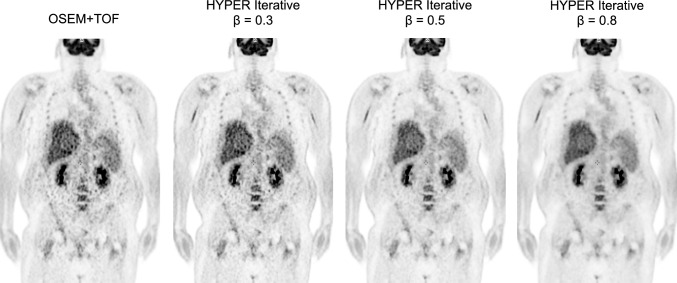
Effects HYPER Iterative in a 59-year-old male with BMI = 41.3. Higher β values result in increased image noise reduction while maintaining contrast

The γ parameter plays a crucial role in the penalty function of HYPER Iterative that is modeled on global and local factors, as well as the regularization strength of β (Fig. 5) [91]. The global factor, defined by the NEC adjusts the image noise level according to changes in injected activity or acquisition duration, compensating for statistical image noise in PET images with low counts. This allows maintained quantitative accuracy while achieving consistent image noise characteristics across images with different numbers of counts. The local factor accounts for spatial variations at the voxel level, correcting for sensitivity variations due to system geometry and attenuation across the field of view (FOV). Consequently, noise distribution can be homogenized within the axial FOV. The regularization strength β is the only user-adjustable parameter and it ranges from 0 to 1. However, in United Imaging Healthcare’s latest PET/CT scanners, such as the uMI Panorama [93], the upper limit of β has been extended to 100. Higher β values result in stronger smoothing, while lower values allow for more iterations without noise control (Fig. 6). This allows users to fine-tune the image quality according to their institutional preferences. The γ parameter in HYPER Iterative corresponds to the β value in Q.Clear. However, the β values in Q.Clear and HYPER Iterative are not directly equivalent because HYPER Iterative also accounts for counts and spatial variations in PET images, which is important to understand. Furthermore, HYPER Iterative incorporates TOF and PSF corrections and automatically adjusts the number of iterations to ensure full convergence. Unlike OSEM, where noise reduction is applied by filtering after reconstruction, HYPER Iterative integrates noise reduction into the reconstruction process, adjusting noise levels at each iteration.

#### Key technical aspects of HYPER Iterative

**Fig. 7:**
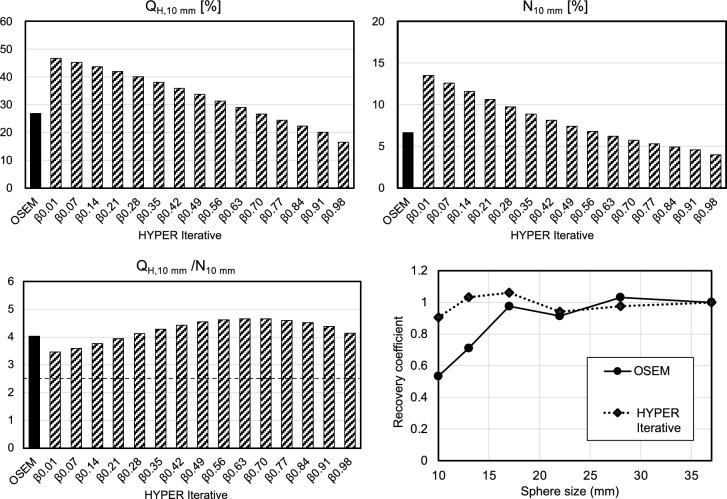
Basic physical characteristics of HYPER Iterative evaluated using NEMA body phantom. Physical characteristics of %contrast Q_H,10 mm_, background variability (N_10 mm_), image quality metric (Q_H,10 mm_/N_10 mm_), and recovery coefficient were assessed

**Fig. 8:**
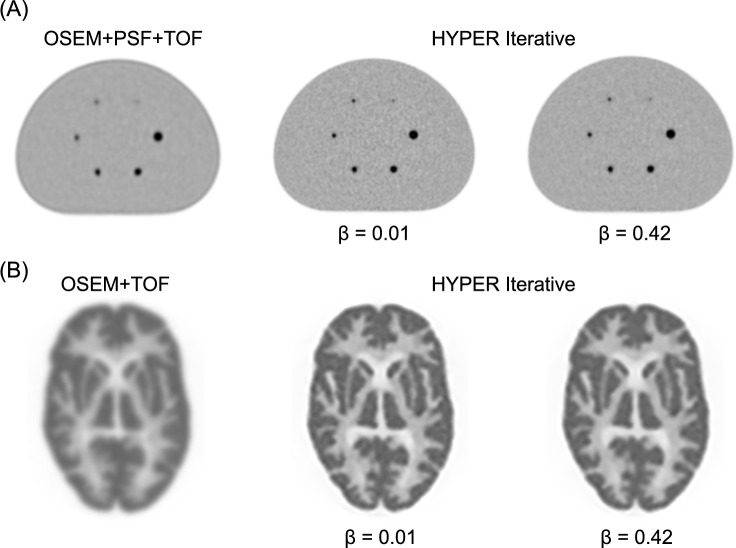
Spatial resolution comparison of OSEM and HYPER Iterative using phantoms. **A** Modified NEMA phantom containing 4, 5, 6, 8, 10, and 13 mm spheres. **B** Hoffman phantom. OSEM conditions for (**A**) was OSEM + PSF + TOF with 3 iterations, 20 subsets, no smoothing and OSEM + TOF with 4 iterations, 20 subsets, 5 mm post filter (non-local means and Gaussian filter [Smooth3]) for (**B**)

We evaluated the fundamental characteristics of HYPER Iterative using a NEMA body phantom with a uMI550 PET/CT system (United Imaging Healthcare) [94, 95]. The background activity concentration was 2.53 kBq/mL, with a sphere-to-background activity ratio of 4:1. We conducted a physical assessment by measuring %contrast (Q_H,10 mm_), background variability (N_10 mm_), image quality indexes (Q_H,10 mm_/N_10 mm_), and recovery coefficients [9]. Increasing the β value resulted in lower contrast but improved image noise characteristics (Fig. 7). The Q_H,10 mm_/N_10 mm_ was maximal at β = 0.63 in HYPER Iterative. The higher recovery coefficient of HYPER Iterative compared with OSEM, even for 10-mm spheres, resulted in superior quantitation accuracy. We acquired PET images of Hoffman and micro-sphere phantoms with a sufficient acquisition duration to visually assess spatial resolution. The spatial resolution was noticeably higher for HYPER Iterative with a lower β value than OSEM (Fig. 8). An acrylic spherical bead phantom (diameter: 4 or 8 mm) designed to replicate the heterogeneous distribution of ^18^F-FDG found that the stability of radiomic features was enhanced by HYPER Iterative compared with OSEM [96].

The results of technical evaluations of HYPER Iterative have recently been published. Comparative studies of phantoms and clinical findings of ^68^ Ga-PET images have found that HYPER Iterative achieves both higher quantitative accuracy and SNR than OSEM [92, 97, 98]. This suggested that HYPER Iterative might reduce injected activity or shorten the duration of PET image acquisition. Combining small voxels (1 or 2 mm) with HYPER Iterative improves contrast and the image noise characteristics of small lesions and β values of 0.8–0.9 yielded the highest contrast-to-noise ratio (CNR), making them suitable for lesion detectability [99]. The fundamental characteristics of HYPER Iterative in ultra-low activity ^18^F-FDG PET imaging of the total body (0.37 MBq/kg) were particularly beneficial for patients who were overweight [100]. However, the authors also noted that extremely high β values could reduce SUV and the visibility of some small lesions, and concluded that β values of 0.3–0.5 were optimal. HYPER Iterative enables higher-quality PET imaging with a shorter scan time than OSEM [101]. The authors reported that HYPER Iterative achieved higher SUV_max_ and TBR, particularly for small lesions, emphasizing its suitability for quantifying low-dose ^18^F-FDG images. They also revealed enhanced visualization of small metastatic lesions in lung cancer by HYPER Iterative compared with conventional OSEM, thus enabling effective early imaging using ^18^F-FAPI total-body PET images at 10 min after radiotracer injection [102]. Based on these findings, HYPER Iterative is considered to offer improved lesion detectability, higher quantitative accuracy, and the potential to maintain image quality even with shorter scan times or reduced radiotracer doses [103].

## Summary of BPL reconstruction

This section introduces studies on BPL methods that have been actively investigated worldwide. The MAP-EM algorithm, which is the basis of BPL methods, was proposed nearly 3 decades ago. However, its implementation in commercial PET systems was hindered by cost, computational demands, design complexity, artifacts, and clinical applicability. Consequently, BPL methods were not widely adopted in clinical practice. However, advances in computing power along with hardware and software innovations have facilitated the clinical use of BPL methods. The application of PET as an imaging biomarker has attracted attention, and BPL methods align with these demands having high image quality and accurate quantitation. A thorough understanding of the characteristics of BPL is essential for optimizing PET imaging, and further clinical studies should strengthen the evidence supporting its value in clinical practice.

## Basic principles of deep learning-based PET reconstruction

Deep learning-based image reconstruction in PET can be broadly categorized into direct and hybrid deep learning-based reconstruction approaches [12, 104]. Direct deep learning-based reconstruction generates PET images from data acquired in a single step, bypassing conventional physical, mathematical, or statistical modeling [105–107]. This approach enables rapid image reconstruction, but requires extensive training datasets and has limited generalizability to variations in tracer distribution and noise levels. Hybrid deep learning-based reconstruction integrates deep learning into iterative reconstruction frameworks. This includes approaches that incorporate deep learning into specific reconstruction steps, and deep learning to model prior or penalty functions or reformulate conventional iterative reconstruction as a convolutional neural network (CNN) [108, 109]. Compared with direct deep learning-based reconstruction, hybrid approaches typically require fewer training data sets and are more adaptable to various imaging conditions. Although not strictly classified as deep learning-based reconstruction, post-processing deep learning methods are often included in this category [12]. Traditional image noise reduction techniques such as Gaussian filtering apply smoothing uniformly across reconstructed PET images, potentially reducing lesion contrast. In contrast, post-processing deep learning methods learn the transformation process between high and low quality PET images, enabling targeted image noise reduction while preserving lesion detail. Post-processing deep learning techniques remain the predominant approach to improving the quality of clinical PET images. Table 2 provides an overview of five deep learning-based PET reconstruction methods currently found in clinical PET systems.

**Table 2:** Deep learning-based image reconstruction methods available on clinical PET systems

	Manufacturer	Type of reconstruction	Model	PET Tracers	Supported PET System(s)
SubtlePET	Subtle Medical	Post-processing noise reduction	Residual + 2.5D U-Net	^18^F-FDG, ^18^F-amyloid, ^18^F-DOPA, ^18^F-Choline, ^18^F-DCFPyL, ^68^ Ga-DOTATATE, ^68^ Ga-PSMA	Vendor-neutral
AiCE	Canon Medical Systems	Post-processing noise reduction	Residual + DCNN	^18^F-FDG	Cartesion Prime
HYPER DLR	United Imaging Healthcare	Post-processing noise reduction	Residual + Dense + 2.5D U-Net	^18^F-FDG	uMI 550, uMI 780, uEXPLORER
HYPER DPR	United Imaging Healthcare	Hybrid	Feedback Net + 2.5D CNN	^18^F-FDG	uMI 550, uMI 780, uEXPLORER, uMI Panorama
Precision DL	GE HealthCare	Post-processing for non-TOF PET image	Residual + 3D U-Net	^18^F-FDG	Omni Legend

## Advanced reconstruction algorithms using deep learning-based PET reconstruction

### SubtlePET

SubtlePET (Subtle Medical) and approved by the FDA in 2019, is one of the earliest deep learning-based image processing technologies for PET [110]. The algorithm reduces noise by applying post-processing to reconstructed PET images. SubtlePET is a vendor-neutral commercial software, it can be integrated into PET/CT systems from various manufacturers. Supported radiotracers include ^18^F-FDG, ^18^F-labeled amyloid, ^18^F-DOPA, ^18^F-choline, ^18^F-DCFPyL, ^68^ Ga-DOTATATE, and ^68^ Ga-PSMA. The deep learning model implemented in SubtlePET uses 2.5D U-Net encoder–decoder architecture that inputs multiple adjacent 2D PET slices and outputs 2D images with suppressed noise [110, 111]. The encoder extracts relevant features from the input slices, and the decoder reconstructs the output through up-sampling of these features. Skip connections integrate low-level feature maps from the encoder into the decoder, which helps to preserve fine structural details. Models are trained on paired datasets consisting of high-noise, low-quality PET images and corresponding high-quality images acquired over prolonged acquisition durations. Instead of generating full images directly, the model learns the residuals between low- and high-quality images, which allows more efficient denoising.

**Fig. 9:**
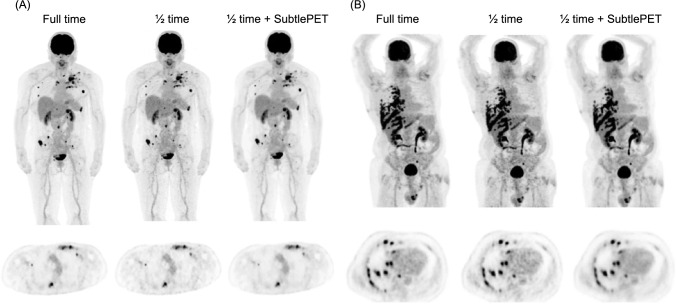
Axial attenuation-corrected and MIP PET images reconstructed with full-time, half-time, and half-time acquisitions with SubtlePET. **A** Female patient with breast cancer weighing 60 kg (BMI, 26.7). B Male patient with lung cancer weighing 98 kg (BMI 32.4). (Reprinted with modification from Bonardel et al. [113]. Copyright © 2022, Bonardel et al., licensed under CC BY). BMI, body mass index; MIP, maximum intensity projection

Recent clinical evaluations of SubtlePET indicate that scan durations or administered doses can be reduced to ~ 50% of current standard protocols [112–115]. The tracer dose for ^18^F-FDG PET can be reduced to ~ 66% of the standard level using SubtlePET, resulting in an estimated 15%–20% reduction in tracer costs [112]. SubtlePET improved the image quality of ^18^F-FDG PET images acquired from persons with a high BMI. It is suitable for clinical practice with a 50% reduction in the amount of time required to acquire images or the dose without compromising diagnostic confidence (Fig. 9) [113]. Image quality was preserved and lesions were detected on ^68^ Ga-PSMA-11 PET images by SubtlePET within 50% the standard acquisition time [56]. However, the SUV_max_ of lesions was substantially altered after processing. Thus direct comparisons between images processed by SubtlePET and conventional reconstruction should not be recommended. Tumor response classifications have been evaluated using SubtlePET to process ^18^F-FDG PET/CT images acquired from 110 patients who underwent baseline and followup assessments [116]. Combining standard and SubtlePET-denoised images across time points revealed that classifications in 10% of patients differed and that these differences could have influenced clinical management in 2%.

### Advanced Intelligent Clear-IQ Engine (AiCE)

**Fig. 10:**
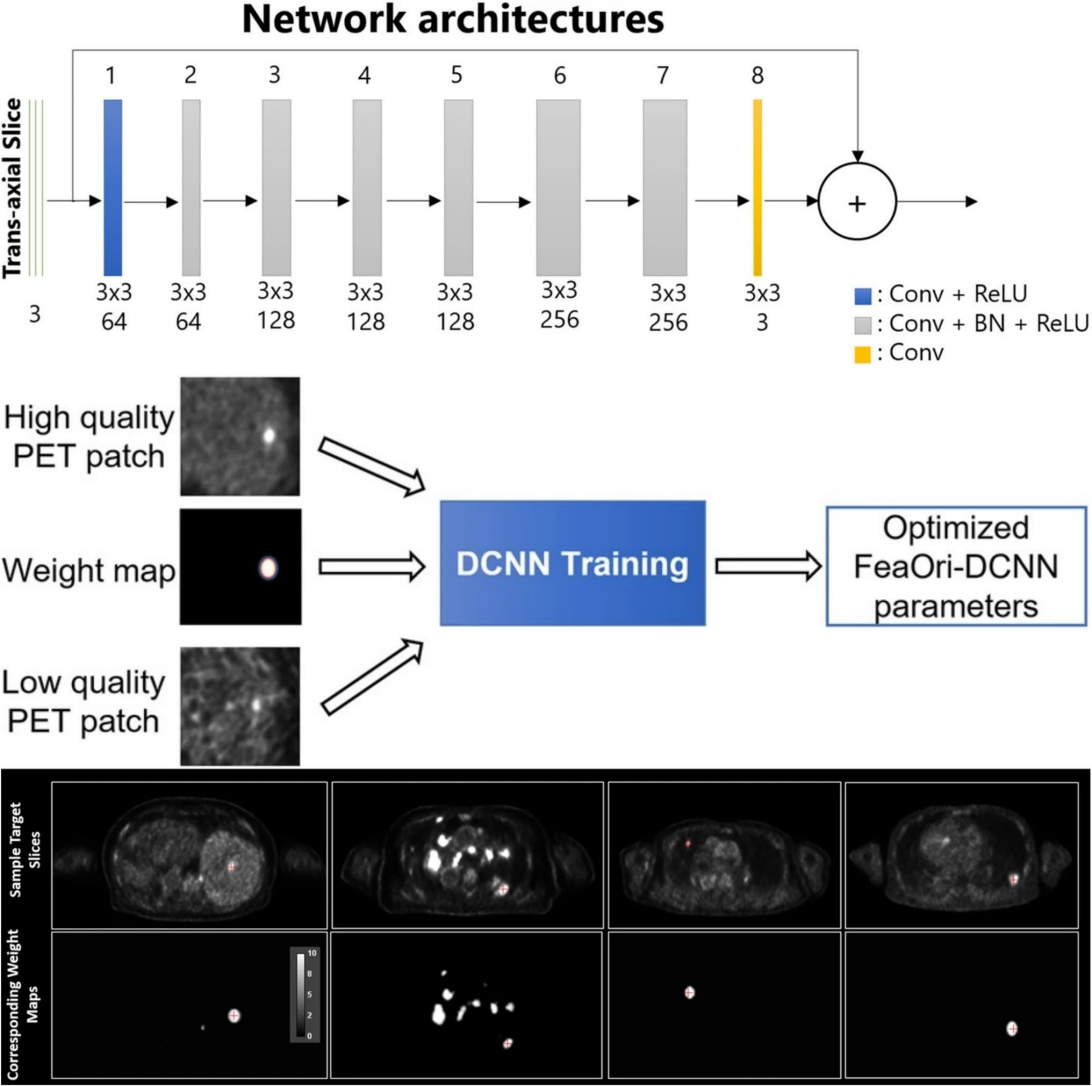
Model architecture of AiCE and weighted map concept. A weighted map highlights regions requiring contrast preservation while training. Only PET images are used as input in final AiCE processing. (Reprinted with modification from Tsuchiya et al. [119]. Copyright © 2023, Tsuchiya et al. licensed under CC BY). AiCE, Advanced intelligent Clear-IQ Engine

The AiCE is a deep learning-based image processing technology that is applicable to PET, CT [117] and MRI [118]. This review focuses on its value to PET, in which AiCE reduces noise in reconstructed ^18^F-FDG PET images and improves overall image quality [119]. On a Cartesion Prime PET/CT system (Canon Medical Systems, Tokyo, Japan), AiCE can be applied to PET and CT using models dedicated to each modality. The AiCE model architecture consists of an eight-layer 2.5D deep convolutional neural network (DCNN) with residual connections, with three consecutive PET slices as input (Fig. 10) [119]. The network is trained using paired low- and high quality PET images with substantial and minimal noise, respectively. Instead of directly generating the denoised image, the model learns the residuals between the low- and high quality images. However, DCNN-based denoising is at risk of excessive smoothing, which can reduce lesion conspicuity and overall contrast. To address this, AiCE introduces weighted maps during training, assigning a weight of 10 to tumors and 1 to background areas. This approach selectively suppresses noise while preserving fine structural details. These weighted maps are applied only during training, as the actual AiCE processing requires only PET images as input.

**Fig. 11:**
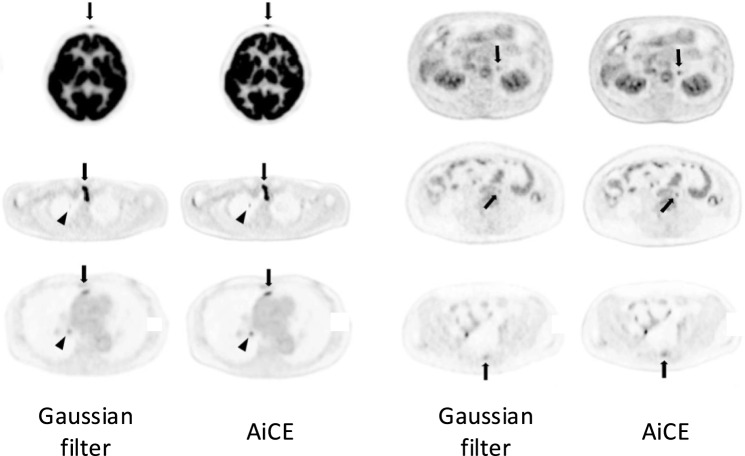
Comparison of ^18^F-FDG PET images processed with Gaussian filter *versus* AiCE. Arrows indicate lesions. Compared with Gaussian filter, AiCE combines noise reduction with improved lesion contrast, which facilitates small lesion detection [120]. (Reprinted with modification from Yamagiwa et al. [120]. Copyright © 2023, Yamagiwa et al., licensed under CC BY)

The key advantages of AiCE-based ^18^F-FDG PET clinical evaluations are robust noise reduction and lesion contrast preservation. While Gaussian filtering reduces SUV_max_ and SUV_peak_, AiCE suppresses noise more effectively without compromising quantitative accuracy [120]. Obvious noise reduction in the liver contributes to improved image uniformity, but Gaussian filtering can obscure subtle accumulations visible with AiCE (Fig. 11). An assessment of ^18^F-FDG PET imaging for breast cancer found that AiCE improved both image quality and quantitation, and that the ability to diagnose axillary lymph nodes was comparable to that of conventional reconstruction [121]. Although modern digital PET/CT systems already provide high-quality images, AiCE might still offer added clinical value.

### uAI HYPER deep learning reconstruction (HYPER DLR)

**Fig. 12:**
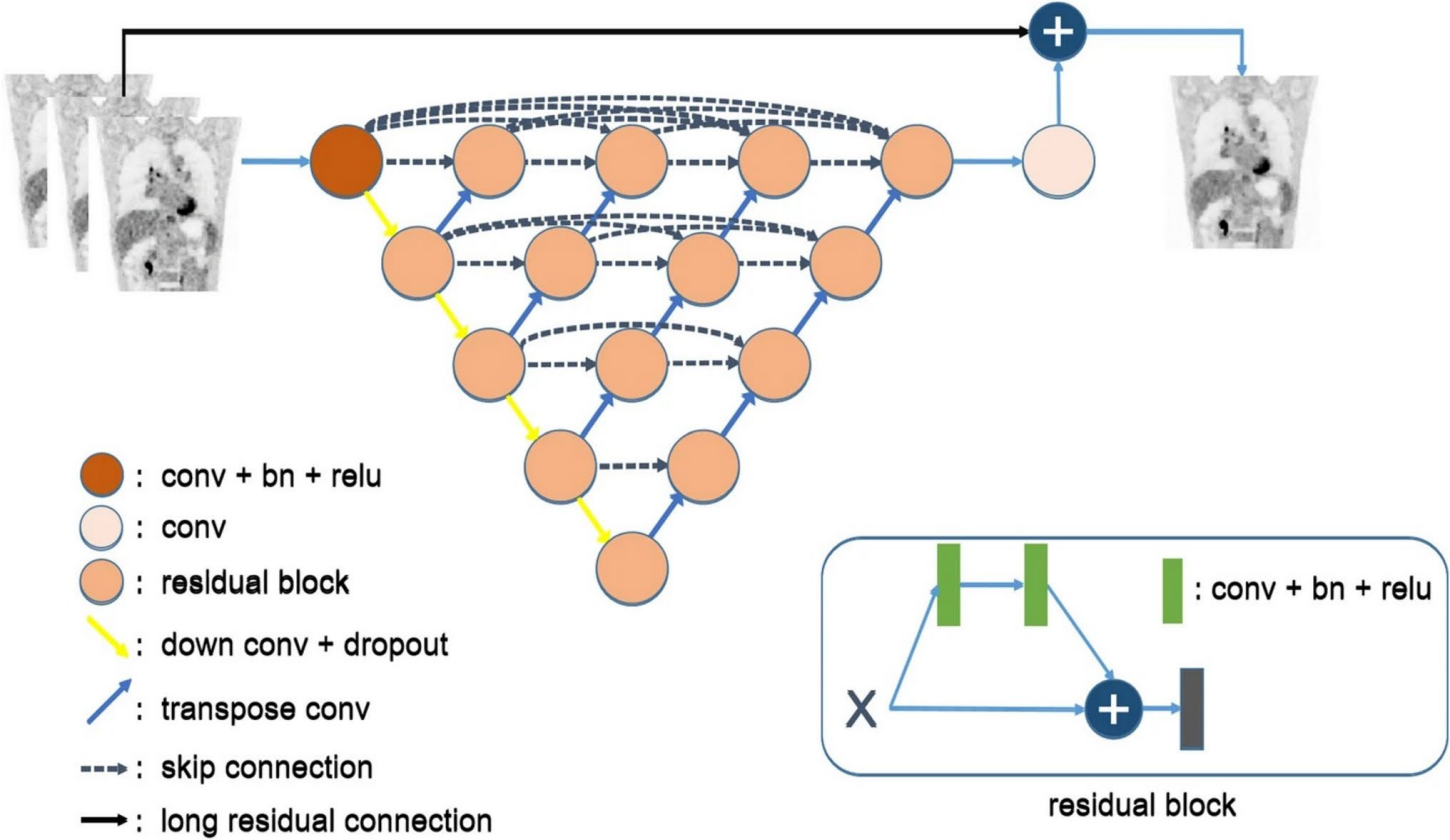
HYPER DLR model architecture. Upper layers incorporate dense connections to preserve high-resolution information, whereas lower layers use sparse connections to balance computational complexity and efficiency. (Reprinted with modification from Xing et al. [122]. Copyright © 2022, Xing et al., licensed under CC BY)

HYPER DLR is a deep learning-based denoising method for reconstructed ^18^F-FDG PET images [122]. The algorithm uses a residual learning approach that estimates the difference between low- and high-quality images. The Head and Body models are dedicated to brain and whole-body PET imaging, respectively. The deep learning model used in HYPER DLR is a 2.5D U-Net +  + architecture incorporating ResNet and DenseNet blocks. The network generates one output PET slice from five consecutive input slices [122] (Fig. 12). The model was trained using 313 paired datasets, where low-quality images were reconstructed from half-duration acquisitions, and high-quality images were acquired at 90–180 s/bed depending on the body region.

**Fig. 13:**
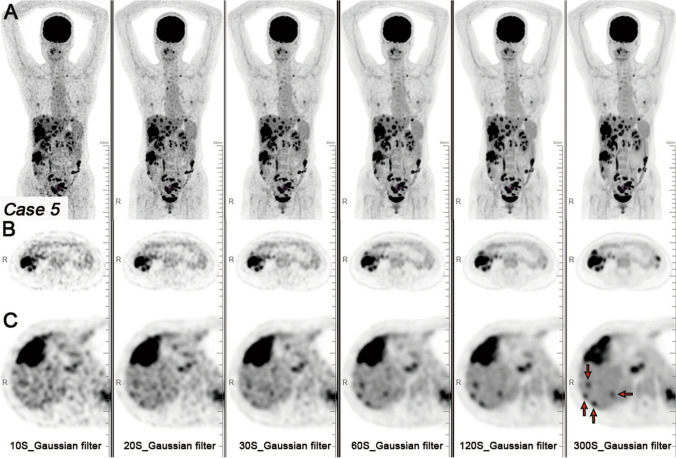
MIP and axial PET images of colon cancer and liver metastases. Axial PET images of (**A**) colon cancer, (**B**) liver metastasis acquired at 10, 20, 30, 60, and 120 s were reconstructed with HYPER DLR, which substantially reduced image noise and improved image quality (**C**). Compared with the 300-s Gaussian-filtered image (reference), sub-centimeter liver metastases (red arrow) appear blurred in HYPER DLR images. (Reprinted with modification from Liu et al. [123]. (Copyright © 2022, Liu et al. licensed under CC BY)

The administered dose was reduced to 25%–50% of the standard during clinical assessments without compromising image quality or quantitative accuracy [122]. Image noise reduction was particularly notable in patients with a high BMI because it led to a 50% dose reduction. Subjective evaluations of lesion contrast tended to favor Gaussian-filtered images over those processed with HYPER DLR. This was probably due to the operator being more familiar with Gaussian smoothing. The authors noted that diagnostic confidence in HYPER DLR images might improve with experience. Scanning could be reduced to 20 s for colorectal and hepatic metastases without affecting quantitative accuracy (Fig. 13) [123]. When PET images were reconstructed with HYPER DLR from 120-s acquisitions, the quality of images was comparable to that of Gaussian-filtered images from 300-s scans. HYPER DLR mitigates the effects of shortened acquisition times that generally increase noise and can lead to elevated SUV_max_ values. However, small liver metastases might be more difficult to detect when the acquisition duration is too short.

### uAI HYPER deep progressive reconstruction (HYPER DPR)

**Fig. 14:**
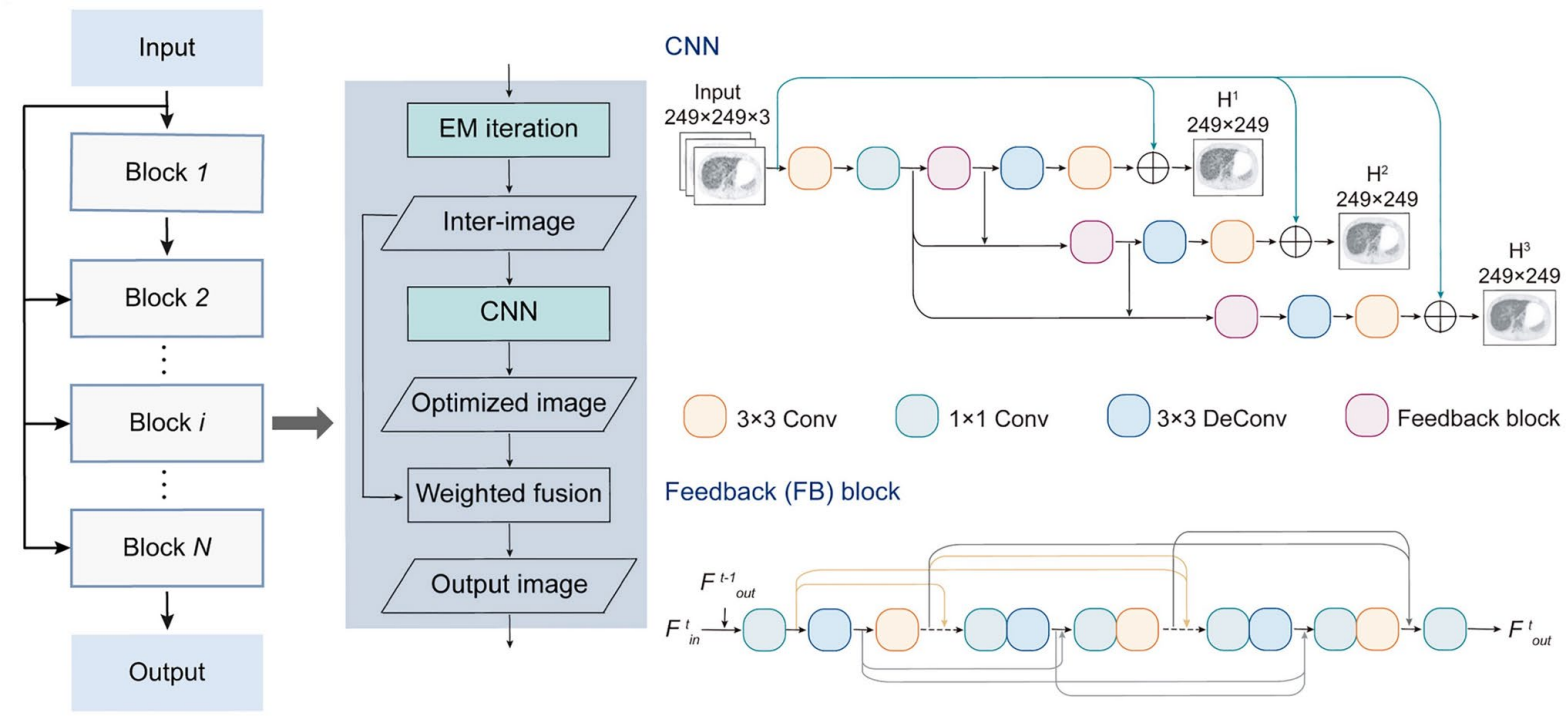
HYPER DPR architecture. Alternating input images pass through CNN-DE and CNN-EH blocks. Both networks use a feedback-block design. (Reprinted with modification from Liao et al. [124]. Copyright © 2023, Liao et al. licensed under CC BY)

HYPER DPR is a hybrid deep learning-based iterative reconstruction method [124]. Unlike SubtlePET, AiCE, and HYPER DLR that operate as post-processing denoising algorithms, HYPER DPR integrates deep learning directly into the iterative reconstruction process. The method includes two CNN modules within the reconstruction loop. One is a denoising network (CNN-DE) that suppresses noise and the other is an enhancement network (CNN-EH) to preserve edges and improve contrast [124, 125] (Fig. 14). After generating an initial image using OSEM, CNN-DE and CNN-EH are alternately applied, with each iteration blending the previous image and the CNN-processed output. Both networks adopt a 2.5D CNN architecture with a feedback mechanism, where intermediate outputs are reintroduced as inputs to enrich feature representation [125]. Weight sharing is also applied to reduce computational load. The model was trained on PET data acquired using the uEXPLORER PET/CT system (United Imaging Healthcare) with axial FOV of 194 cm with 900-s acquisitions. The CNN-DE was trained on image pairs from short- and long-duration acquisitions to model noise reduction across count numbers. The CNN-EH was trained on early- and late-iteration reconstructions to guide contrast recovery. The combination of these networks within the reconstruction loop of HYPER DPR is an advanced AI-based approach to high-quality PET image reconstruction via simultaneous noise suppression and contrast restoration.

**Fig. 15:**
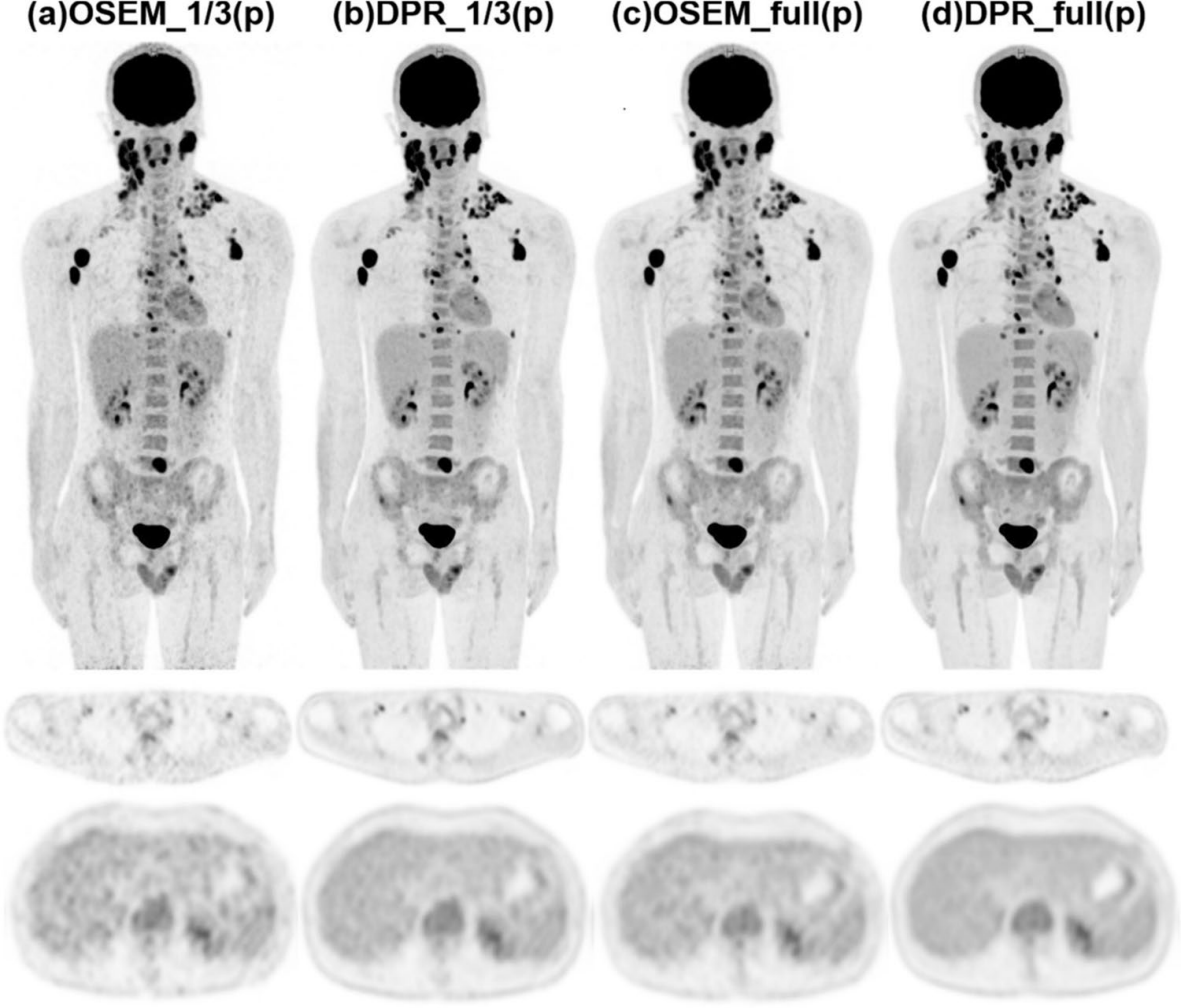
Images of 31-year-old male patient diagnosed with Hodgkin’s lymphoma, reconstructed using HYPER DPR and OSEM. We reconstructed PET data using both algorithms at full and one-third acquisition durations to enable a comprehensive comparison. Image noise is reduced and edge definition is enhanced in ^18^F-FDG PET images reconstructed by HYPER DPR compared with OSEM at the same acquisition time. (Reprinted with modification from Wang et al. [126]. Copyright © 2022, Wang et al. licensed under CC BY)

HYPER DPR improves small-lesion contrast and detection performance, even when the tracer dose is reduced to one-third of the standard amount, while maintaining quantitative reliability and uniformity in background regions such as blood and muscle (Fig. 15) [126]. Furthermore, HYPER DPR preserved image quality and Deauville scoring accuracy of images acquired from patients with lymphoma under conditions of a low dose or a short acquisition period, thus supporting its clinical applicability to protocols with reduced exposure to activity [127]. A comparative study of patients with lung cancer found that HYPER DPR resulted in higher SUV_max_, SUV_mean_, and TBR, along with lower metabolic tumor volumes compared to OSEM, particularly in solid nodules [128]. However, as with other next-generation reconstruction methods, the SUV_max_ of lesions is often higher when reconstructed with HYPER DPR than conventional OSEM. This discrepancy can impact established clinical criteria, underscoring the need to update quantitative standards as PET technology evolves.

### Precision DL

**Fig. 16:**
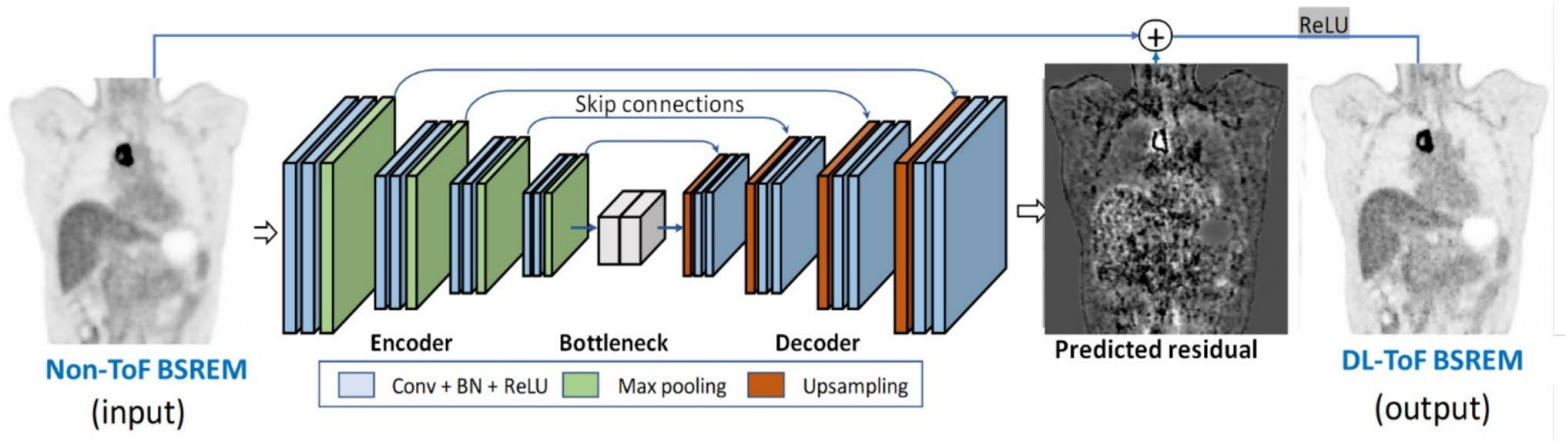
Precision DL architecture. Residual learning is used to estimate differences between images without and with TOF. Final output is generated by adding this residual to the non-TOF image. (Reprinted with modification from Mehranian et al*.* [129]. Copyright © 2022, Mehranian et al. licensed under CC BY)

Precision DL is a deep learning-based reconstruction method designed to learn the difference between PET images with and without TOF, rather than simply denoising [129]. This method is applied to PET data reconstructed without TOF to approximate the benefits of TOF information, and generate TOF-like PET images. The network architecture is based on a 3D residual U-Net (Fig. 16) [129] trained to predict residuals between images with and without TOF. Precision DL is trained on Q.Clear images reconstructed with various penalization β factors. Low (β ≈ 960), medium (β ≈ 450), and high (β ≈ 335) parameter settings increase the amount of contrast enhancement at the cost of reducing noise suppression. After PET image acquisition, users can select a β value for Q.Clear and specify the output strength of Precision DL as low (LPDL), medium (MPDL), or high (HPDL), depending on the clinical objective. As the output strength of Precision DL increases from low to high, noise levels, SBR, and SNR also increase [130].

**Fig. 17:**
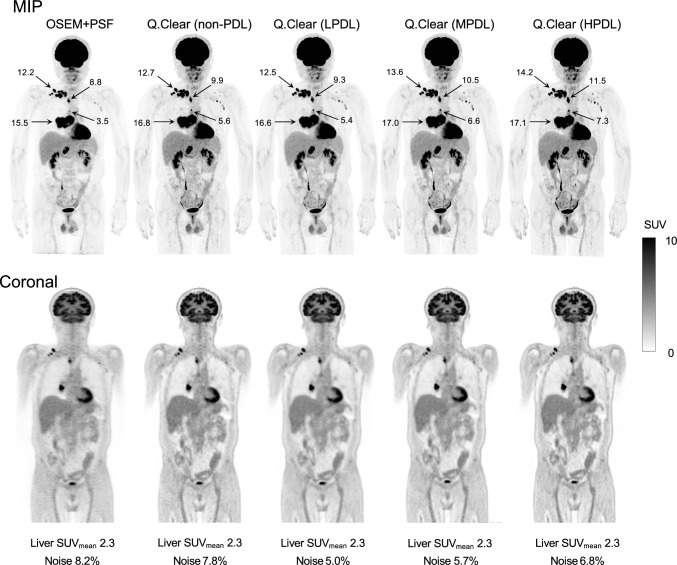
Non-TOF ^18^F-FDG PET images processed with varying strength of Precision DL. Applying Precision DL improves noise characteristics while maintaining quantitative accuracy. Increasing the Precision DL intensity further enhances lesion contrast. (Reprinted with modification from Miwa et al. [132]. Copyright © 2023, Society of Nuclear Medicine and Molecular Imaging)

Initial clinical reports regarding Precision DL have been published [129, 131, 132]. The findings suggest that Precision DL is particularly useful for lesion detection in regions with high background uptake, such as the liver or mediastinum. Precision DL can reduce attenuation correction artifacts near the diaphragm, which are often visible in PET images reconstructed without TOF [129]. This effect might be attributed to its ability to approximate the spatial consistency provided by TOF information [133]. A recent study [134] and the Precision DL white paper from GE HealthCare [135] have further validated its ability to improve image quality and replicate TOF-specific features in clinical and phantom settings, supporting its robustness across various imaging conditions. We recently evaluated the effects of Precision DL on ^18^F-FDG PET images acquired using the Omni Legend 32 digital BGO-based PET/CT system (GE HealthCare) [130]. We found that Precision DL reduced noise and background variability while increasing SUV_max_ for small lesions, particularly in high-background regions such as the liver and mediastinum (Fig. 17) [130, 132]. A combination of Q.Clear and Precision DL further improved SUV_max_ and image quality compared with OSEM + PSF. Q.Clear with a β value of 500–600 combined with MPDL is recommended for oncologic PET/CT imaging with the Omni Legend [130]. While Precision DL was initially applicable only to ^18^F-FDG, recent advances have extended its application to other tracers such as ^18^F-PSMA, ^68^ Ga-PSMA, and ^68^ Ga-DOTATATE PET for theranostic imaging [4, 136].

### Future challenges and summary of deep learning-based PET image reconstruction

Deep learning technologies continue to rapidly evolve. Diffusion models have attracted attention because of their success in image synthesis, and are thus being explored for PET applications [137, 138]. Removing noise and other artifacts from PET images using denoising diffusion probabilistic model (DDPM) is feasible and surpasses U-Net and GAN-based approaches [139]. Although diffusion models require substantial computational resources, recent GPU advances have enabled fully 3D PET image denoising [140]. Beyond denoising, diffusion-based PET image reconstruction methods have been proposed [141, 142] that introduce data consistency constraints within the diffusion sampling process using trained DDPM. While still at an early stage, diffusion-based reconstruction offers superior image quality and flexibility, indicating its potential for future clinical applications.

Advances in PET instrumentation are further expanding opportunities for integration with deep learning-based processing [143]. Deep learning can directly estimate TOF from raw detector waveforms [144–146], potentially replacing conventional pipelines based on electronics. Moreover, total-body PET systems are producing ultra-high-sensitivity images [147], and generating near noise-free data that serve as ideal training sets for both denoising and reconstruction networks [148].

## Data Availability

The data set used and/or analyzed during the current study are available from the corresponding author on reasonable request.
